# Age-Related Effects on Future Mental Time Travel

**DOI:** 10.1155/2016/1867270

**Published:** 2016-04-07

**Authors:** Filomena Anelli, Elisa Ciaramelli, Shahar Arzy, Francesca Frassinetti

**Affiliations:** ^1^Department of Psychology, University of Bologna, 40127 Bologna, Italy; ^2^Fondazione Salvatore Maugeri Hospital IRCCS, 46042 Castel Goffredo, Italy; ^3^Centro Studi e Ricerche in Neuroscienze Cognitive, 47023 Cesena, Italy; ^4^Neuropsychiatry Lab, Faculty of Medicine, Hadassah Hebrew University Medical School, 91200 Jerusalem, Israel; ^5^Department of Neurology, Hadassah Hebrew University Medical Center, 91200 Jerusalem, Israel

## Abstract

Mental time travel (MTT), the ability to travel mentally back and forward in time in order to reexperience past events and preexperience future events, is crucial in human cognition. As we move along life, MTT may be changed accordingly. However, the relation between re- and preexperiencing along the lifespan is still not clear. Here, young and older adults underwent a psychophysical paradigm assessing two different components of MTT: self-projection, which is the ability to project the self towards a past or a future location of the mental time line, and self-reference, which is the ability to determine whether events are located in the past or future in reference to that given self-location. Aged individuals performed worse in both self-projection to the future and self-reference to future events compared to young individuals. In addition, aging decreased older adults' preference for personal compared to nonpersonal events. These results demonstrate the impact of MTT and self-processing on subjective time processing in healthy aging. Changes in memory functions in aged people may therefore be related not only to memory per se, but also to the relations of memory and self.

## 1. Introduction

Healthy aging is associated with changes in autobiographical episodic memory, that is, the ability to recall one's past experiences in their spatial and temporal context [1, 2]. In a seminal study, Levine and colleagues tested memory for past experiences in younger and older adults using the Autobiographical Interview (AI), which allows quantifying separately the episodic (internal) and semantic (external) details constituting participants' autobiographical reports. While external details were comparable across groups, internal details were significantly fewer in older compared to younger adults, indicating that aging is associated with a loss in autobiographical memory specificity [3]. More recently, Addis and colleagues demonstrated that the loss in specificity characterizing older adults' autobiographical memory spreads to their ability to imagine future events ([4]; see also [5, 6]). Using a modified version of the AI, young and older adults were required to remember past events and imagine plausible future events. Older (compared to young) adults produced fewer internal details and more external details for both past and future events, suggesting a similar effect of aging on remembering the past and imaging the future (e.g., [4, 7]).

The parallel performance in remembering the past and imagining the future in older adults dovetails with functional neuroimaging (fMRI) evidence that imagining the future engages a distributed network of brain regions largely overlapping with those activated while remembering the past [8–10]. One way of conceptualizing the neural overlap between remembering the past and imagining the future rests on the “constructive episodic simulation hypothesis” [11], according to which the episodic memory system supports the construction of future events by extracting and recombining flexibly information stored in memory into a novel event. In light of this hypothesis, the problems in imagining the future observed in older adults may derive from their difficulties at remembering past events: if autobiographical memories contribute to the construction of future events, then poor autobiographical memories will result in poor simulations of the future. This is confirmed by neuroimaging data showing a reduced activity in older compared to younger adults in both conditions [11].

Despite evidence that older adults performed worse in both remembering the past and imagining the future, more recently Gaesser and colleagues [12] found that they might show even more pronounced difficulties in imagining future events than in remembering past events. In this study, indeed, older adults' difficulties at imagining the future remained significant when controlling for memory (as well as narrative) performance. As for the anatomical bases of this age difference, a recent fMRI study by Addis and colleagues showed that whereas young adults engaged ventrolateral prefrontal and frontopolar cortex more strongly while imagining future events than when remembering past events, older adults did not show the same asymmetry. This may be due to an inability in modulating prefrontal cortex activity in response to the increased constructive demands of the future task [13]. Additionally, in recent studies, older adults had more difficulties at imagining future events than fictitious, atemporal events compared to younger adults [14], and were less capable of using future thinking adaptively [15]. Thus, older adults' difficulties with episodic future thinking are not completely explained by a general deficit with imagining complex experiences (such as fictitious, atemporal events) [16] or with travelling in time mentally (such as while remembering the past) [12].

There is great interest in revealing the component processes of future thinking that are especially susceptible to the effect of aging. In this respect, humans' ability to recall the past and anticipate the future has been conceptualized as “mental time travel” (MTT), that is, travelling mentally back and forward in time in order to reexperience past events and preexperience future events [6, 17–19] or “self-projection” [20]. Recently, it was proposed that humans spatially map events in real and imagined past and future on an imagined time line, the mental time line (MTL) ([21]; for a review see [22]). Self-projection, therefore, may be conceived as the ability to imagine oneself located in a specific point on the MTL. Arzy and colleagues have proposed that, “projecting” one's habitual self-location in time to the past or future, humans not only recall and predict events, but also change their mental egocentric perspective on life events [23, 24]. Indeed, when we assume a different location in time, life events are regarded differently with respect to their relations to past or future: if we project ourselves back to 15 years ago, for example, last year's events are future (i.e., relative future) events, whereas they were past events if seen from the present time.

Arzy and colleagues [23] developed a psychophysical MTT paradigm requiring young participants to “project” their self-location in time to the past, the present, or the future (i.e., self-projection). Then, participants were required to determine whether a given event happened before (relative past) or might happen after (relative future) the specific self-location they had assumed in time. This classification of events is called self-reference. Behaviorally, the main findings were that response times (RTs) and error rates (ERs) were (1) higher in past and future self-locations than in the present self-location, highlighting the costs of projecting one's self in time, (2) lower for relative future than for relative past events, suggesting that humans are “tuned” to the future as opposed to the past, and (3) lower for personal compared to nonpersonal events, indicating that self-relevant information is prioritized. At the neural level, these effects activated both regions traditionally involved in autobiographical memory and regions associated with space processing and self-representation, including the anteromedial temporal, inferior frontal, and temporoparietal cortices [23, 24].

To specify the potential changes of the mental time travel in aging, we tested young and older adults in a modified version of the MTT paradigm from Arzy et al. [23]. First, basic episodic/autobiographic memory abilities were assessed by asking young and older adults to classify events as past or future from the perspective of the present time, that is, the one that is typically adopted in laboratory studies. To assess self-projection, we asked subjects to make similar self-referencing judgments from a past or future self-location in time. We hypothesized that older adults may face difficulties in (1) classifying events as past or future (self-reference) or (2) adopting a past or future perspective (self-projection). Considering that prefrontal activity is downregulated during future mental time travel in older compared to young adults [13], we predicted older adults would especially face problems at projecting themselves in the future time.

## 2. Materials and Methods

### 2.1. Participants

Twenty-four healthy young adults (8 males, mean age ± sd: 24 ± 1.11 years old) and twenty-four healthy older adults (13 males, 67 ± 7.57 years old) participated in the experiment. There was no statistical difference between the two groups in the level of education (young group = 16.5 years; elderly group = 14.7 years; *p* = 0.31). All participants were right-handed and had normal or corrected-to-normal vision and no history of neurological or psychiatric diseases.

Participants were naive as to the purpose of the study and provided written consent to participate in the experiment, which was approved by the Ethical Committee of the University of Bologna, in agreement with the 2008 Helsinki Declaration. Additionally, older adults did not show any cognitive impairment as measured by the Mini Mental State Examination (MMSE score ≥ 29) [25] or an impairment of executive function as measured by the Time and Weight Estimation Test (STEP total score > 40) [26].

### 2.2. Stimuli and Procedure

Participants sat in front of a 15-inch color monitor, at a distance of about 60 cm. Brief descriptions of personal (e.g., car license and first child) or nonpersonal events (e.g., Obama's election and Middle East peace) were presented on the computer screen (for a complete list of stimuli, see Table 1). Most stimuli (events) we used have already been used and validated in previous studies [23, 24], whereas some other events have been adapted according to the elapsed time. In particular, nonpersonal events were the same for young and older adults. Past nonpersonal events comprehended very famous events from the last 40/50 years of national and international history, and future nonpersonal events involved events that may happen in the future but whose timing cannot be predicted with certainty.

With respect to personal events, these were, in all cases, events characterizing important stages of one's lives, but the specific events were adapted to the two groups, with the main aim of having equally important and meaningful events for younger and older adults. Past personal events were referred to the last 20 and 50 years, for young and older people, respectively, in proportion to their past life duration. Future personal events comprehended for young as well as old people events that could happen in the next 30 years or whose timing cannot be predicted with certainty.

**Figure 1:**
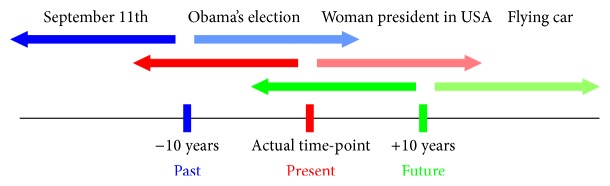
Stimuli and procedure. Participants were required to project themselves in three different self-locations in time (past, present, or future) and determine whether the event being presented was located in the past or the future relative to the current self-location.

For each event, participants were required to indicate if the event had already happened (relative past event) or was yet to happen (relative future event). Participants performed the task in three different conditions, which corresponded to three different self-locations in time (Figure 1). In one condition, they were required to answer the questions while imagining themselves as being located in the present time (present self-location), in a second condition they had to answer while imagining themselves as being located in the past (10 years ago, past self-location), and in a third condition they had to answer while imagining themselves as being located in the future (10 years from now, future self-location). Thus, in each self-location condition, participants had to determine whether the event being presented was located in the past or the future relative to the current location of the self in time.

Each self-location condition included 24 stimuli, half personal and half nonpersonal, equally distributed between relative past and relative future events, which were presented in random order for a total of 72 trials. Each event appeared in the center of the computer screen and remained visible until a response was given, with an interstimulus interval of 1000 ms. Judgments were given using the index finger of the left or right hand if the event was past or future, respectively (counterbalanced). E-Prime 2.0 software was used for stimulus presentation and response collection. Before the experimental task, subjects performed a brief practice session, with 8 stimuli randomly presented. Moreover, the experimenter encouraged participants to self-project in time, for example, focusing on their age in 10 years ago/in 10 years or on the exact year it was/will be 10 year ago/in 10 years.

While nonpersonal events were the same for young and older adults, personal events were adapted for the two groups and therefore differed between groups. An independent group of twenty-five young adults (3 males, mean age: 23 years, age range: 22–28) and twenty-five older adults (10 males, mean age: 66 years, age range: 53–80) rated the events in the main experiment for level of importance (with 1 = completely unimportant event and 5 = very important/life-changing event) and intensity of emotion (with 1 = event eliciting low levels of emotion and 5 = event eliciting high levels of emotion) on a Likert scale. Events presented to the young adults were evaluated by an independent group of young adults whereas events presented to the older adults were evaluated by an independent group of older adults. No statistical difference was found between the two groups (*p* > 0.05 for both comparisons). For each rating, response means were entered into a repeated-measures ANOVA with* Event* (personal and nonpersonal) and* Self-location* (past, present, and future) as within-subject factors and* Group* (young and older adults) as between-subject factor. The analysis on events' importance did not reveal a significant main effect or interaction (all* p* > 0.43). The analysis on intensity of emotion showed a significant main effect of* Event *[*F*_(1, 44)_ = 26.89, *p* < 0.001, power = 0.99], since personal events received higher scores than nonpersonal events (3.84 versus 3.21, resp.). Importantly, neither the effect of* Group* nor that of* Self-location* was significant, suggesting that potential differences in mental time travel between young and older adults could not be attributed to differences in the quality of the events considered by different groups or at different self-locations in time.

The present self-location condition was always run first, because a pilot study revealed that this made the task more easily comprehended by older adults. The past and future self-location conditions were run second or third (order counterbalanced across participants). Participants' performance in the present condition, which did not require self-projection in subjective time, was considered as a pure measure of episodic/autobiographical memory, as it required locating in time (past versus future) autobiographical as well as nonpersonal events. Next, to examine age-related changes in self-projection, response times (RTs) and error rates (ERs) in the present self-location condition were subtracted from those in the past and future self-location conditions. At the end of the experiment, anamnestic data were collected to determine correct responses according to the responses provided separately by each participant.

### 2.3. Data Analysis

A first analysis was conducted to assess participants' general ability to locate autobiographic as well as nonpersonal events in time. To this aim, RTs and ERs (analyses were also conducted combining speed and accuracy by calculating the inverse efficiency score (i.e., IES) [27, 28] which consists of reaction time divided by 1 − proportion of errors. These results confirm those obtained using RTs.) relative to the present self-location condition were analyzed using a repeated-measures ANOVA with* Event* (personal and nonpersonal) and* Response* (relative past and relative future) as within-subject factors and* Group* (young and old) as between-subject factor. Then, a second analysis was carried out to investigate age-related changes in self-projection. For each participant, we calculated the difference between RTs and ERs (analyses were also conducted combining speed and accuracy by calculating the inverse efficiency score (i.e., IES) [27, 28] which consists of reaction time divided by 1 − proportion of errors. These results confirm those obtained using RTs.) obtained in the past and future self-location condition and in the present condition (ΔRTs and ΔERs, resp.). In both cases, a positive Δ indicated a disadvantage for the past/future self-location condition with respect to the present condition (more time needed or more errors occurring while answering questions from the past/future compared to the present self-location), whereas a negative Δ indicated an advantage. These scores were submitted to a repeated-measures ANOVA with* Event* (personal and nonpersonal),* Self-location* (past and future), and* Response* (relative past and relative future) as within-subject factors and* Group* (young and old) as between-subject factor. Post hoc Duncan tests were performed on significant interactions.

## 3. Results

### 3.1. Older People Have Difficulty in Processing Personal Events

**Figure 2:**
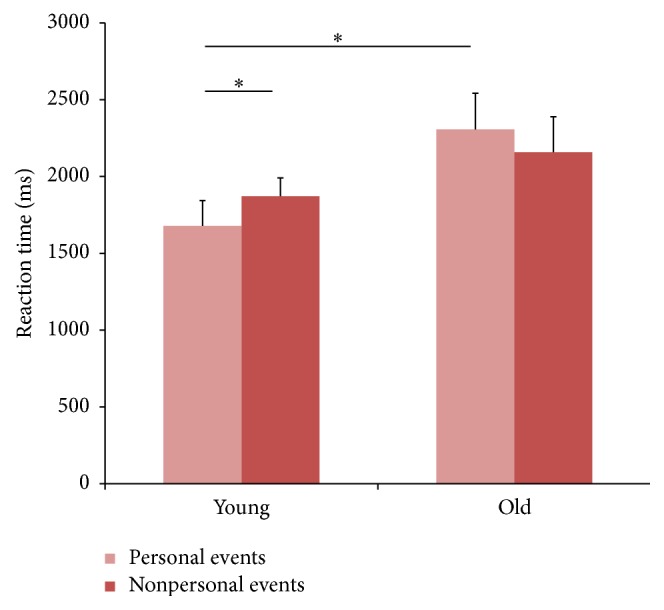
*Group* ×* Event* interaction on RTs. Values are in ms and error bars depicted SEM. Asterisks indicate significant differences (*p* < 0.05).

Analysis of RTs revealed a significant interaction between* Group* and* Event* [*F*_(1, 46)_ = 5.74, *p* < 0.01, power = 0.76] (Figure 2). Duncan post hoc tests showed that young adults responded faster to personal than to nonpersonal events (1677 versus 1871 ms, resp., *p* < 0.05), whereas older adults showed comparable RTs for personal and nonpersonal events (2306 versus 2158 ms, resp., *p* = 0.10). Moreover, a difference between groups emerged only with personal events, since young adults responded faster than older adults (*p* < 0.01), whereas no difference between groups was found in nonpersonal events (*p* = 0.16).

Analysis of ERs did not show any significant main effect or interaction (all *p* > 0.09).

These results indicate that older adults are slower than young adults in determining whether personal/autobiographical events are past or future, whereas they perform normally while judging nonpersonal events, thus showing a difficulty in autobiographical memory.

### 3.2. Older People Have Difficulty in Projecting Themselves to the Future

**Figure 3:**
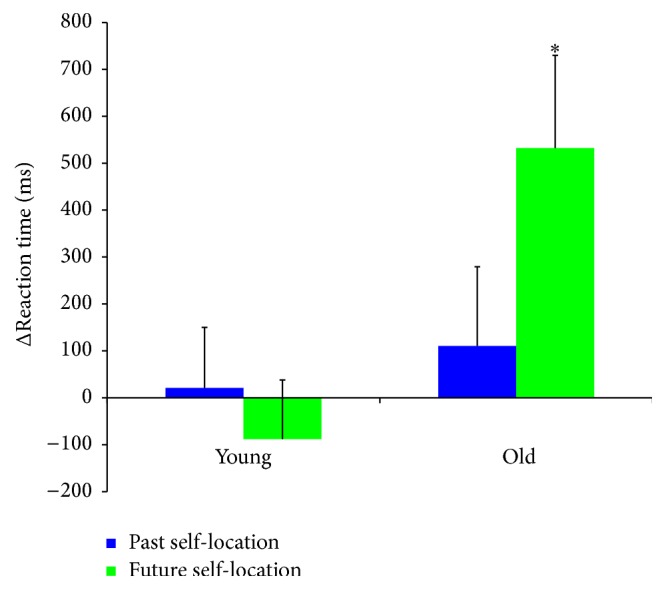
*Group* ×* Self-location* interaction on ΔRTs. Values are in ms and error bars depicted SEM. Asterisk indicates significant differences (*p* < 0.05).

The analysis on ΔRTs showed a significant main effect of* Group* [*F*_(1, 46)_ = 8.13, *p* < 0.01, power = 0.80] and a significant* Group* ×* Self-location* interaction [*F*_(1, 46)_ = 16.59, *p* < 0.001, power = 0.98] (Figure 3): in the future self-location, older were disproportionately slower than young adults (532 versus −88 ms, resp., *p* < 0.001), whereas no difference in ΔRTs was recorded between older and young adults in the past self-location condition (110 versus 21 ms, resp., *p* = 0.53). Moreover, older adults showed a disadvantage (larger ΔRTs) in evaluating events from a future compared to a past self-location (532 versus 110 ms, resp., *p* < 0.001), whereas young adults reported comparable ΔRTs in both conditions (−88 versus 21 ms, *p* = 0.24). The three-way interaction among* Group* ×* Self-location* ×* Event* was also significant [*F*_(1, 46)_ = 14.24, *p* < 0.001, power = 0.96]. Post hoc tests revealed that the disadvantage observed for older adults in the future (versus past) self-location condition was driven by personal events. Indeed, when judging personal events, older adults showed significantly larger ΔRTs from the future versus past self-location (750 versus −139 ms, resp., *p* < 0.001), whereas younger adults did not (future 9 versus past 2 ms, *p* = 0.94). In contrast, when considering nonpersonal events, older adults did not show the same disadvantage in the future versus past self-location (315 versus 360 ms, resp., *p* = 0.63), and young adults were even faster in the future self-location condition (future −185 versus past 40 ms, *p* < 0.05).

The analysis on ΔERs showed a significant interaction between* Group* and* Response* [*F*_(1, 46)_ = 7.42, *p* < 0.01, power = 0.76], as ΔERs for future responses were higher in elderly than in young participants (6% versus −1%, resp., *p* < 0.01). No group difference was found for past-responses (young 2% versus older adults 2%, *p* = 0.80).

Taken together, these results indicate that after controlling for basic autobiographic/episodic memory performance (present self-location), older compared to younger adults were slower to judge personal events from the future self-location and made more errors while making relative future than relative past responses (in both the future and past self-location).

## 4. Discussion

This study revealed difficulty in future processing in older adults on various planes. Older adults were significantly slower compared to young adults in judging personal events while adopting a future self-location perspective. Since older participants did not perform worse than young adults while adopting a past self-location, our findings indicate that aging does not affect self-projection in time in general but, rather, projecting one's self to the future. Consistent with this, in a recent study Gaesser and colleagues [12] asked younger and older adults to remember past events, imagine future events, and describe a complex picture of a natural scene in as much detail as possible. In older compared to young adults, internal details were reduced across all conditions. However, older adults' performance at imagining the future remained significantly worse even after controlling for both description and memory performance. This finding suggests that older adults may face problems in processes necessary for future thinking that go beyond those involved in autobiographical memory and description of complex scenes.

It is important to emphasize that older adults' preserved self-projection towards the past suggests that age differences in self-projecting towards the future cannot be explained with poor comprehension of task demands. Nor can older adults' difficulties with future self-projection be attributed to future self-projection being inherently more difficult than past self-projection: in fact, young adults were faster at classifying events from the future compared to the present self-location (negative Δ), whereas the past self-location made them slower (positive Δ). Rather, the observed age-related effect may relate to differences in the cognitive processes underlying self-projection towards the future versus past time. First, more executive resources may be required for self-projection toward the future compared to the past, as while the past has already happened, the future is a mental construction [29]. Projecting the self 10 years to the future requires simulating how one's self, others, and the world will be in 10 years. Second, older adults' difficulties at projecting themselves in the future may be related to an inability to construct and use self-related information, which is required to conceive the personal future [30, 31]. D'Argembeau and Mathy [32], for example, have shown that self-knowledge, especially knowledge about one's own goals, is particularly important to frame search and integration of information for future thinking.

A selective difficulty in processing the future in older adults also emerged with respect to self-reference, which is the ability to determine whether events are located in the past or future in reference to a given self-location. In both the past and future self-location, older adults made more errors than younger adults in judging events belonging to the relative future, whereas no group differences emerged for relative past events. Similar effect of MTT with respect to past events in both groups despite differences in the presented stimuli suggests that this difference does not account for the identified effect with respect to future processing. It has been proposed that the primary role of MTT is the anticipation of future occurrences and decision-making related to the future (e.g., [33]), leading humans to perform better for future-related events [23]. One possibility, therefore, is that poorer performance measured in older participants in this task may be linked to a loss of such future-preference. This result may be related to a tendency with aging to be (adaptively) less motivated and less oriented towards the future (see also [15]). When people perceive time as limited, indeed, they shift priorities from acquisition-related goals to maintenance or loss-prevention (e.g., socioemotional selectivity theory) [34]. Notably, the loss of future-preference was also found in the past self-location condition, that is, when older adults were contemplating events that were not actually future (i.e., with respect to the present time) or necessarily yet to happen.

Finally, unlike young adults [23, 24], older adults did not show a “self-effect,” that is, a faster performance with personal compared to nonpersonal events. The self-effect in young adults relates to several evidence that autobiographically relevant information boosts both memory encoding and retrieval (see also [35, 36]). Participants' ratings indicated that personal and nonpersonal events did not differ in importance, but they differed in the emotions they elicited, which were more intense for personal events. This was expected, given that personal events are strongly connected to one's self and identity, and connected to past desires and future goals [37]. Older adults, too, judged personal events as more emotionally laden than nonpersonal events, but they showed a reduced ability to locate them in time. This finding may be interpreted as an age-related decline in the access to contextual details of autobiographical memories (e.g., [3, 4]), due to changes in the efficiency of strategic retrieval processes with aging (e.g., [1, 38]).

While the present work does not include neuroimaging, neuroanatomical information may enrich the scope of our results. Previous work has suggested that both autobiographical memory and future thinking rely on a distributed network of brain regions, including medial temporal and frontal cortices, posterior cingulate cortex, retrosplenial cortex, and lateral parietal and temporal areas [4, 23, 24, 39, 40]. These regions grossly overlap with a “default network” of brain regions whose activity is enhanced by internally focused thought [39, 41–43]. Our study highlights a change in autobiographical memory in older adults, consistent with the observed decline with age in the functionality of the default network [44]. Considering that older adults especially face difficulties in self-projection towards the future and self-reference to future events and that prefrontal cortex is preferentially involved in imagining future events [13], it may be speculated that the results observed here are to be mainly ascribed to age-related changes in the structure and function of prefrontal cortex (e.g., [1, 45–48]). However, more posterior regions of the default network, affected, for example, in Alzheimer's disease [42], may also play a role in explaining future-thinking changes with aging.

## 5. Conclusion

To conclude, our results show that aging impacts self-projection to the future as well as reference of the self to future events and occurrences. These findings suggest that changes in mental time travel in healthy aging (and possibly even in pathology) may be related not only to memory functions per se, but also to the relations between self and memory. Considering that the ability to envisage the future has a strong adaptive value, for example, allowing considering the potential consequences of choices (e.g., [33]), losing the capability to travel in time towards the future may have important consequences for older adults' psychological well-being and decision-making. In this perspective, the present findings may be relevant to interpret the behavioral difficulties observed in aging as well as pathological states such as mild cognitive impairment, Alzheimer's disease, and other dementia states.

## Figures and Tables

**Table 1:** List of events presented to young and older participants.

	Past self-location	Present self-location	Future self-location
Young participants: personal events (relative past)	First best friend	First time at the dentist	Graduate
	First school trip	10th birthday	30th birthday
	First time at the sea	First school day	Maturity examination
	Bicycle without wheels	First political vote	First political vote
	10th birthday	Maturity examination	First salary
	First day of school	Driving license	Driving license

Young participants: personal events (relative future)	30th birthday	30th birthday	Living on the moon
	First political vote	First son	Retirement
	First son	Silver wedding	Silver wedding
	Maturity examination	Son marriage	Son marriage
	Leave the hometown	Graduate	Son graduate
	Driving license	First salary	50th birthday

Older participants: personal events (relative past)	First boyfriend	Using glasses	Using glasses
	First car	40th birthday	80th birthday
	First salary	First son	Silver wedding
	Driving license	First car	First grandchild
	40th birthday	Retirement	First trip by train
	First day of school	First hospitalization	Golden wedding

Older participants: personal events (relative future)	80th birthday	80th birthday	Living on the moon
	Son retirement	First great grandchild	Son retirement
	First great grandchild	Grandchild marriage	Diamond marriage
	Grandchild marriage	Diamond wedding	Grandchild marriage
	Admission to the nursing home	Admission to the nursing home	Flying to Mars
	Golden wedding	Son retirement	100th birthday

Young and older participants: nonpersonal events (relative past)	Pertini's election	Obama's election	Obama's election
	Fall of Berlin wall	First use of euro	First use of euro
	Chernobyl disaster	Chernobyl disaster	Europe unites
	Man on the moon	Man on the moon	Gaddafi's death
	September 11th	September 11th	Pope Francesco's election

Young and older participants: nonpersonal events (relative future)	Pope Francesco's election	Peace in middle east	Flying car
	Gaddafi's death	Completely defeat illnesses	World peace
	Woman president in USA	Woman president in USA	Completely defeat mafia
	Rita Levi Montalcini's death	Completely defeat mafia	Completely defeat illness
	End of the world	End of the world	End of the world
	Completely defeat mafia	Completely defeat world hunger	Completely defeat world hunger
